# Opening our eyes to Global Health; a philosophy of universal values

**DOI:** 10.1007/s40037-015-0210-z

**Published:** 2015-09-14

**Authors:** Val Wass

**Affiliations:** Faculty of Health, Keele University, ST5 5BG Staffordshire, UK

**Keywords:** Global health, Self-awareness, Diversity, Professional values

## Abstract

Globalization is advancing at a pace. As we strive to introduce ‘Global Health’ into clinical curricula we risk fundamental misunderstandings unless we clearly define what we aim to achieve. Clinicians must be prepared for a life time of uncertainty, change and challenge. The fluctuating world arena will undoubtedly impact on their future work in ways we cannot predict. Population migration, climate change and shifts in cultural dominance are already at play. Global health risks being translated through the eyes of Western ideology as disease-based curricula focused paternalistically on ‘helping’ the developing world. We must not lack humility to open eyes to learning within the context of increasingly diverse environments and patient populations. Global health is as ‘local’ as it is ‘international’. It should be viewed, I argue, as a philosophy based on the values and expectations found within ourselves and our communities. Responding to globalization lies not only in knowledge but embraces human rights, justice and, most importantly, self-awareness. Knowledge is more easily translated into curriculum objectives. We risk letting future clinicians and their patients down if we ignore the other universal values.

The only true voyage of discovery…, would be not to visit strange lands but to possess other eyes, to behold the universe through the eyes of another… [1]

My own personal voyage of discovery began when I set off as a relatively mature senior lecturer in general practice to undertake the Masters in Health Professions Education (MHPE) in Maastricht in the Netherlands. This impacted profoundly on my views on global health. It was a true eye opener as I learnt to ‘behold the universe’ through the eyes of others. This led me to discover myself in ways which positively changed my life, impacted on my views of medical education and stimulated research which has contributed to an emerging conception of the issues we face.

Globalization is advancing at a pace bringing to the fore many challenges for medical education as both health care workers and patients become increasingly ethnically diverse and internationally mobile. The Lancet report ‘Health Professions for a New Century’ highlights the need to ‘transform education to strengthen health care systems in an interdependent world’ [2]. The authors raise concerns over our potential failure to prepare our health professionals for tomorrow’s world. We need to step back and realign our curricula to address the inequities of health in a global context. The Flexnerian disease-based concepts [3] still tend to dominate. Traditional knowledge and disease-based factual approaches to global health within the curriculum are easily conceptualized. Learning in a multiprofessional, multi-ethnic group in Maastricht taught me that there is far more to understanding global health than knowledge alone.

Perhaps the most profound eye opener was facing my own personal values. Working with sixteen international health worker students (only three doctors), from four continents (with the exception of Australia) was a profoundly moving experience. As a middle-aged woman becoming a student again was a true culture shock; living in student accommodation, no secretary and information technology naivety all lifted me well out of my comfort zone. The greatest challenge, though, was facing my own competitive nature which had been pervasive throughout medical training and my strong Western paternalistic values. It was a humbling experience working as a Problem-Based Learning group and becoming immersed in the multifactorial dynamics of contrasting personalities, cultural values and inter-professional tensions. We bonded too; the communal social experiences in the evenings, collective help to face travel and the bitter biting winter cold and developing a mutual understanding of the challenges faced by many when they returned home. I vividly remember sitting alongside Agnes, a nurse from Tanzania, on a very stark, basic dormitory bed, weeping with her as she described and lamented the loss of life in her village as AIDs, at its peak, was sweeping through all generations. Quite unforgettable. Everything I learnt about universal global values was generated from seeing the world through the eyes of my colleagues within the classroom, not from visiting strange lands.

It is time to move global health forward. Indeed, I would argue, unless we are clear what we mean by ‘global health’ we risk taking a simplistic approach to its interpretation and implementation. It is perhaps not surprising that conceptualizing and embedding ‘global health’ within clinical training is challenging for educators. The problems we face have been aptly verbalized: ‘*We live in a world where change is exponential and we are helping to prepare students for jobs that don’t yet exist, using technologies that have not yet been invented, in order to solve problems that we don’t know are problems yet!*’ [4] We recognize that students need to be prepared for ‘*a lifetime of uncertainty, change, challenge and emergent or self-created opportunity*’ [4] but are unable to imagine just what the emerging challenges might be. The changes I have witnessed in clinical management since I trained have been immense and totally unpredictable. When I was a medical student the hospital wards were full of patients who after a heart attack were immobilized on total bed rest for 28 days. Now they have interventions to repair coronary arteries within hours and are home and active the next day. The learning environment in which I trained has been totally transformed in a way none of us could have predicted.

We have an obligation to prepare our trainees for this uncertain future yet can only guess what the implications might be. Several factors are already at play. Technologies for knowledge transfer move rapidly forward. Inevitably climate change will increasingly challenge health care delivery globally. We already experience some of the impact; London is experiencing its hottest day ever as I write [5]. Cultural dominance is shifting inevitably away from the West as both China and India exponentially expand in many ways [6]. Viewing the issue of global health with Western values may not be appropriate. Paternalistic Western values, and firmly held beliefs that our way of doing things is the best, are difficult to overcome. In his book ‘Turning the World Upside Down’ Lord Nigel Crisp encapsulates this. ‘*If we are really interested in improving global health, then current Western practices are wrong: we export our ideology—that the state and professionals know best—and we import their doctors, nurses and health professionals to work in our health care systems. What we should be doing is the opposite: we should import their growing awareness that health is created by citizens and communities and we should export our best people to share some of our technical expertise*’ [7]. I cringe when medical students, not yet qualified even for supervised practice, view their elective experiences as an opportunity ‘to help’ and not ‘learn from’ a developing country. There is even an expectation they can use these international opportunities to practise clinical skills on patients at a level we would not condone for patients at home in the West. Time and time again I see colleagues, when working to develop curricula and assessments internationally, offering opportunities for them to witness and emulate how we do it in the UK. They fail to allow them to develop more creative and culturally appropriate local methods from which we in the West might learn and move forward. We are not harnessing the opportunities globalization offers.

As the ethnic diversity of our students, health care workers and, above all, patients increases, there are many opportunities to advance our understanding and learn within the West itself. Yet we fail to embrace these. Research shows that educators remain fixed in stereotype [8]. Despite expanding cultural diversity within the student body, there are difficulties in sharing and learning from each other [9]. There appear to be significant barriers to sharing personal cultural values and a failure for students of different ethnicity to integrate [10]. A perception that global health addresses ‘disease’ rather than ‘health’ pervades. I recall a quote from some research we did where a student framed it as ‘*African sleeping sickness in microbiology; that’s kind of cultural awareness isn’t it? Rare and dodgy diseases like that’* [9]. Yet, arguably, to embrace the fundamental aims of global health, the priorities lie with improving and achieving equity in *health for all* people worldwide. To achieve this understanding the issues lie as much within individuals in our own institutions and learning environments as externally in the international arena.

Richard Horton, Editor in Chief of the Lancet, frames the dilemma eloquently. He argues, with passion, that global health should be viewed as a philosophy which embraces ‘people, human rights, justice and knowledge’ [6]. Universal values of equity, dignity, respect and humility must be fostered within the individual; not just the knowledge. These values lie within ourselves and are consequently difficult to frame as intended learning curriculum outcomes. We focus on the patient and develop generic patient-centred communication frameworks for understanding them. Yet students, as the evidence suggests [11], need support in sharing and understanding their own values and prejudices if they are to communicate with cultural sensitivity. The doctor-patient interaction is not a one-way street. We fail as educators to cultivate self-awareness in students or to empower them to develop the resilience and ability to cope with the uncertainties of globalization and unpredictable challenges ahead. This dilemma may have been around for some time. Ian McWhinney said in 1997: *We can only attend to a patient’s feelings and emotions if we know our own, but self-knowledge is neglected in medical education* [12].

If we are to truly foster global health as a philosophy of universal values, then as educators we must help our students to discover themselves, just as I discovered and realigned my values and prejudices on the MHPE. If trainees are to work effectively in an increasingly diverse and changing world, international experience and visiting ‘strange lands’ is not a requirement to open their eyes to this. The opportunities exist within our own culturally diverse learning environments where the universal values underpinning the philosophy of global health pervade. We risk letting future clinicians and their patients down if we ignore these.
